# Ultrasonographic Knee Abnormalities and Their Association with Pain in Young Male Handball and Basketball Athletes: A Cross-Sectional Study

**DOI:** 10.3390/diagnostics16132134

**Published:** 2026-07-07

**Authors:** Nicoleta Anamaria Pascalau, Alexandru Bogdan Ilieș, Brigitte Osser, Csongor Toth, Gyongyi Osser, Laura Ioana Bondar, Gheorghe Codruț Bulz, Anca Maria Sabău, Mihaela Gavrila-Ardelean, Corina Dalia Toderescu

**Affiliations:** 1 Department of Psycho Neuroscience and Recovery, Faculty of Medicine and Pharmacy, University of Oradea, 410087 Oradea, Romania; nicoleta.pascalau@didactic.uoradea.ro (N.A.P.); ilies.alexandrubogdan@student.uoradea.ro (A.B.I.); 2 Doctoral School of Biomedical Sciences, University of Oradea, 410087 Oradea, Romania; toth.csongor1@student.uoradea.ro; 3 Faculty of Physical Education and Sport, “Aurel Vlaicu” University of Arad, 310130 Arad, Romania; gyongyi.osser@uav.ro; 4 Master’s Programme in Management of Musculoskeletal Disorders, Faculty of Medicine and Pharmacy, University of Oradea, 410087 Oradea, Romania; 5 Department of Biology and Life Sciences, Faculty of Medicine, “Vasile Goldiș” Western University of Arad, 310025 Arad, Romania; bondar.laura@uvvg.ro; 6 Department of Physical Education, Sports and Physiotherapy, Faculty of Geography, Tourism and Sport, University of Oradea, 410087 Oradea, Romania; bulz.codrut@uoradea.ro (G.C.B.); asabau@uoradea.ro (A.M.S.); 7 Faculty of Educational Sciences Psychology and Social Work, Aurel Vlaicu University of Arad, 310130 Arad, Romania; mihaela.gavrila@uav.ro; 8 Faculty of Pharmacy, “Vasile Goldiș” Western University of Arad, 310045 Arad, Romania; toderes-cu.corina@uvvg.ro

**Keywords:** athletes, basketball, diagnostic ultrasound, handball, knee injuries, musculoskeletal diseases, pain measurement, patellar tendinopathy, sports medicine, ultrasonography

## Abstract

**Background/Objectives:** Knee injuries and overuse-related disorders are common among athletes participating in jumping sports such as handball and basketball. Musculoskeletal ultrasonography is increasingly used for the assessment of knee pathology; however, evidence regarding the prevalence and clinical relevance of ultrasonographic abnormalities in young athletes remains limited. The aim of this study was to investigate the prevalence of ultrasonographic knee abnormalities in young male handball and basketball athletes and to examine their association with pain intensity. **Methods:** A cross-sectional observational study was conducted between June 2025 and June 2026 and included 69 competitive male athletes (35 handball players and 34 basketball players). All participants underwent bilateral knee ultrasonographic examination using a standardized assessment protocol and completed a questionnaire regarding demographic and training characteristics. Knee pain intensity was evaluated using the Visual Analogue Scale (VAS). Comparisons between sports were performed using χ^2^ and *t*-tests, while associations between participant-level ultrasonographic findings and pain were evaluated using independent-samples *t*-tests (or Mann–Whitney U tests, as appropriate), with Cohen’s d effect sizes and exploratory multivariable linear regression. Sensitivity analyses stratified by sport were additionally performed. **Results:** Patellar tendinopathy was the most prevalent ultrasonographic abnormality (21.0%), followed by medial meniscal abnormality (15.9%) and infrapatellar bursitis (13.0%). Athletes with patellar tendinopathy, medial meniscal abnormality, or infrapatellar bursitis had significantly higher VAS pain scores than athletes without the corresponding ultrasonographic abnormality. Patellar tendinopathy demonstrated the strongest association with participant-reported pain (VAS: 4.1 ± 1.3; Cohen’s d = 1.24; *p* < 0.001). Handball athletes exhibited a significantly higher prevalence of patellar tendinopathy than basketball athletes (34.3% vs. 11.8%; OR = 3.90, 95% CI: 1.09–13.95; *p* = 0.027). In multivariable regression analysis adjusted for age, BMI, sport type, previous knee injury, and weekly training volume, patellar tendinopathy (β = 1.34, *p* < 0.001), medial meniscal abnormality (β = 0.70, *p* = 0.017), and infrapatellar bursitis (β = 0.54, *p* = 0.046) remained independently associated with higher pain scores. The regression model explained 39% of the variance in VAS pain scores (R^2^ = 0.39). **Conclusions:** Ultrasonographic knee abnormalities are common among young male handball and basketball athletes and are significantly associated with pain intensity. Because ultrasonography has limited ability to characterize intra-articular pathology, particularly the menisci, the ultrasonographic abnormalities identified in this study should not be interpreted as definitive diagnoses, and MRI remains the reference imaging modality when comprehensive evaluation of intra-articular pathology is clinically indicated. Patellar tendinopathy was the most prevalent ultrasonographic abnormality and was most strongly associated with pain intensity. These findings support the use of musculoskeletal ultrasonography as a complementary imaging modality alongside clinical assessment in the evaluation of symptomatic athletes. However, prospective longitudinal studies are required to determine whether these ultrasonographic abnormalities have prognostic value for future pain, functional limitation, or time-loss injury.

## 1. Introduction

Participation in competitive sports provides substantial physical, psychological, and social benefits; however, it is also associated with an increased risk of musculoskeletal injuries, particularly among athletes involved in high-impact and jumping sports [1,2,3,4,5]. Knee injuries are among the most common complaints reported by young athletes and may significantly affect athletic performance, training continuity, and long-term joint health [6,7,8]. Sports such as handball and basketball require repetitive jumping, landing, sprinting, abrupt changes of direction, and frequent deceleration movements, all of which place considerable mechanical stress on the knee joint and surrounding soft tissues [9,10].

The knee is particularly vulnerable to overuse and traumatic injuries involving the menisci, tendons, ligaments, and periarticular structures [11,12,13]. Patellar tendinopathy, often referred to as “jumper’s knee,” represents one of the most prevalent overuse conditions in athletes participating in jumping sports and is frequently associated with pain, functional impairment, and reduced sports participation [14,15,16,17]. Similarly, meniscal abnormality, bursitis, and ligament abnormalities may contribute to persistent symptoms and increase the risk of future joint degeneration if not identified and managed appropriately [12,13].

Diagnostic imaging plays a crucial role in the evaluation of knee pathology in athletes. Among available imaging modalities, musculoskeletal ultrasonography has emerged as a valuable, non-invasive, radiation-free, and cost-effective tool for the assessment of superficial soft-tissue structures around the knee [8,18,19,20]. Ultrasound examination allows dynamic visualization of tendons, ligaments, bursae, and periarticular tissues and can be performed rapidly in both clinical and sports medicine settings [8,20]. Moreover, ultrasonography has demonstrated good diagnostic accuracy for several tendon-related disorders and may facilitate the early detection of structural abnormalities before the onset of severe clinical symptoms [21,22,23,24]. Recent advances in musculoskeletal ultrasonography, including elastographic techniques such as shear wave elastography (SWE), have further expanded its role in the assessment of lower-limb musculoskeletal disorders and tendon-related pathology [25].

Although musculoskeletal ultrasonography has become increasingly important in sports medicine, magnetic resonance imaging (MRI) remains the reference imaging modality for the evaluation of intra-articular knee pathology. MRI studies have reported a high prevalence of patellar tendinopathy, meniscal abnormalities, cartilage lesions, and bone marrow edema among athletes participating in jumping sports [26]. Previous MRI investigations involving handball and basketball athletes have consistently identified patellar tendinopathy, meniscal abnormalities, bone marrow edema, and cartilage changes as the most common structural findings, particularly among athletes exposed to repetitive jumping and pivoting movements [27,28,29]. However, MRI is comparatively expensive, less accessible, and not always feasible for routine assessment in sports medicine settings [30,31]. In contrast, musculoskeletal ultrasonography provides a rapid, dynamic, and cost-effective evaluation of superficial soft-tissue structures, although it has recognized limitations in the assessment of deep intra-articular abnormalities, particularly the menisci and articular cartilage [32,33]. Therefore, understanding the extent to which the most common MRI-reported abnormalities can also be identified by musculoskeletal ultrasonography is clinically relevant and may help define the complementary role of ultrasound in the clinical assessment of athletic knee disorders.

Despite the growing use of ultrasonography in sports medicine, important knowledge gaps remain. Previous studies have primarily focused on individual knee disorders, specific athletic populations, or elite-level athletes, often emphasizing imaging findings without simultaneously evaluating their relationship with pain and clinical presentation [34,35,36,37]. Furthermore, relatively few studies have compared ultrasonographic findings between handball and basketball athletes, despite the substantial biomechanical demands shared by these sports. The clinical significance of asymptomatic ultrasonographic abnormalities also remains controversial, as structural changes detected by imaging do not always correlate with pain intensity or functional limitations [3,21].

Understanding the prevalence and clinical relevance of ultrasonographic abnormalities in young athletes may improve clinical assessment and inform future research on the potential role of ultrasonography in the assessment of athletes with knee symptoms. Whether ultrasonographic abnormalities identify athletes who may benefit from closer clinical monitoring requires confirmation in prospective longitudinal studies. Identifying which structural abnormalities are most strongly associated with pain may assist clinicians and sports medicine practitioners in prioritizing diagnostic and therapeutic decisions. In addition, comparing ultrasonographic findings across different sports may provide valuable insights into sport-specific injury patterns and mechanical loading mechanisms.

Therefore, the aim of the present study was to investigate the prevalence of ultrasonographic knee abnormalities among young male handball and basketball athletes and to examine their association with self-reported pain intensity. A secondary objective was to compare the distribution of ultrasonographic findings between the two sports and to identify factors independently associated with pain using multivariable regression analysis.

Based on the existing literature, we hypothesized that: (1) patellar tendinopathy and meniscal abnormalities would be among the most prevalent ultrasonographic findings; (2) athletes with ultrasonographic abnormalities would report higher pain intensity than athletes without detectable ultrasonographic abnormalities; (3) handball athletes would exhibit a higher prevalence of tendon-related abnormalities than basketball athletes; and (4) ultrasonographic abnormalities, particularly patellar tendinopathy, would be associated with higher pain intensity.

## 2. Materials and Methods

### 2.1. Study Design and Participants

A cross-sectional observational study was conducted between June 2025 and June 2026 involving competitive male handball and basketball athletes. Participants were recruited from regional sports clubs and training centers. The study was intentionally designed to include only male athletes in order to investigate a more homogeneous study population and to reduce potential confounding related to known sex-specific differences in knee biomechanics, injury mechanisms, and tendon adaptations.

Athletes were eligible for participation if they met all of the following criteria:

• Male sex;
• Age between 16 and 30 years;
• Active participation in organized handball or basketball training for at least one year;
• Regular participation in training sessions and official competitions during the study period;
• Provision of written informed consent to participate in the study.
• Athletes were excluded if any of the following conditions were present:
• Previous knee surgery within the last 12 months;
• Acute traumatic knee injury preventing participation in regular sports activities at the time of assessment;
• History of systemic musculoskeletal, inflammatory, rheumatologic, or neurological disorders affecting lower-limb function;
• Incomplete clinical, questionnaire, or ultrasonographic data;
• Refusal or inability to provide informed consent.

A total of 69 athletes fulfilled the eligibility criteria and were included in the final analysis. The study population comprised 35 handball players and 34 basketball players.

**Figure 1:**
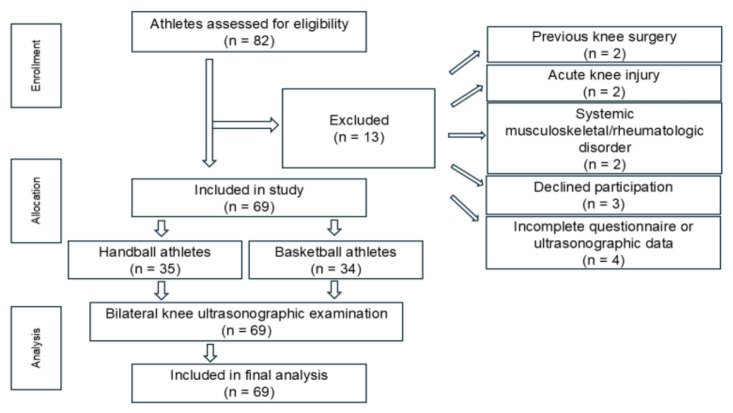
Flow diagram of participant screening, enrollment, and inclusion in the final analysis.

All participants underwent bilateral knee ultrasonographic examination and completed the study questionnaire, including pain assessment using the Visual Analogue Scale (VAS). No participants were lost after enrollment, and complete ultrasonographic and clinical data were available for all included athletes. The participant screening process, eligibility assessment, and inclusion in the final analysis are summarized in Figure 1.

### 2.2. Data Collection and Questionnaire Assessment

Demographic and sports-related information was collected using a standardized questionnaire administered at the time of evaluation. The questionnaire was completed individually by each participant under the supervision of a member of the research team to ensure completeness and accuracy of the recorded information.

The questionnaire included the following variables:

• Age (years);
• Place of residence (urban/rural);
• Dominant lower limb (right or left);
• Sport practiced (handball or basketball);
• Training experience (years);
• Weekly training volume (hours/week);
• Previous history of knee injury.

Anthropometric measurements were obtained according to standardized procedures. Body weight was measured to the nearest 0.1 kg using a calibrated digital scale, and height was measured to the nearest 0.1 cm using a portable stadiometer. Participants were assessed barefoot and wearing light sports clothing.

Body mass index (BMI) was calculated using the following formula:

(1) BMI = weight (kg)/height^2^ (m^2^)

Participants were also asked to report any previous knee injuries diagnosed by a healthcare professional and any episodes of knee pain experienced during sports participation. Information regarding training exposure was collected to characterize the athletic profile of the study population and to identify potential factors associated with ultrasonographic abnormalities and pain intensity.

All assessments were performed during the competitive season under standardized conditions. Data were recorded anonymously and entered into a dedicated database for subsequent statistical analysis.

### 2.3. Ultrasonographic Examination

All participants underwent bilateral knee ultrasonographic examination performed by a physician with extensive experience in musculoskeletal ultrasonography. The examiner routinely performs diagnostic ultrasound assessments in sports medicine and musculoskeletal rehabilitation settings.

Ultrasonographic examinations were conducted using a Samsung HS40 ultrasound system (Samsung Medison, Seoul, Republic of Korea) equipped with a high-frequency linear-array transducer operating at 5–18 MHz. Standardized machine settings were maintained throughout the study, including gain, depth, and focal zone adjustments optimized for superficial musculoskeletal structures. Color Doppler imaging was used when indicated to assess intratendinous vascularity and inflammatory changes.

To reduce potential assessment bias, the ultrasonographer was blinded to participants’ VAS pain scores and questionnaire responses at the time of image acquisition and interpretation.

Participants were examined in a standardized supine position with both knees evaluated according to the technical recommendations of the European Society of Musculoskeletal Radiology (ESSR) for musculoskeletal ultrasound of the knee. The examination protocol included standardized longitudinal and transverse assessment of the patellar tendon, quadriceps tendon, collateral ligaments, infrapatellar bursae, tibial tuberosity region, and accessible medial and lateral meniscal margins, as these structures represent the knee tissues most commonly involved in sports-related overuse and traumatic injuries in jumping athletes. Additional musculotendinous structures, such as the semimembranosus tendon and other posteromedial tendons, were not systematically evaluated because they were outside the predefined scope of the study. Dynamic assessment was performed when necessary to improve visualization of superficial musculoskeletal structures.

Patellar tendinopathy was defined according to established sonographic criteria, including focal or diffuse tendon thickening, hypoechogenicity, loss of normal fibrillar echotexture, and/or intratendinous Doppler signal suggestive of neovascularization. Quadriceps tendinopathy was defined using analogous criteria. Infrapatellar bursitis was identified by abnormal fluid accumulation within the infrapatellar bursa.

Meniscal findings were recorded as ultrasonographic signs suggestive of meniscal abnormality rather than definitive meniscal tears. These findings included irregular meniscal contour, focal hypoechoic clefts, abnormal echogenicity, meniscal extrusion, or focal disruption of the visible meniscal margin. Because MRI remains the reference imaging modality for comprehensive assessment of intra-articular meniscal pathology, ultrasonographic findings were interpreted as ultrasound abnormalities and not as a substitute for MRI confirmation.

To enhance consistency, all examinations were performed by the same investigator using a standardized acquisition protocol. A random sample comprising 15% of the ultrasonographic examinations was re-evaluated by the same investigator after a two-week interval to assess intra-observer reliability. Cohen’s kappa coefficient was calculated for the categorical ultrasonographic findings evaluated in this study, including the presence or absence of patellar tendinopathy, meniscal abnormalities, infrapatellar bursitis, collateral ligament abnormalities, quadriceps tendinopathy, and Osgood–Schlatter changes. The overall intra-observer agreement was excellent (κ = 0.84). No continuous ultrasonographic measurements were included in the reliability assessment.

**Figure 2:**
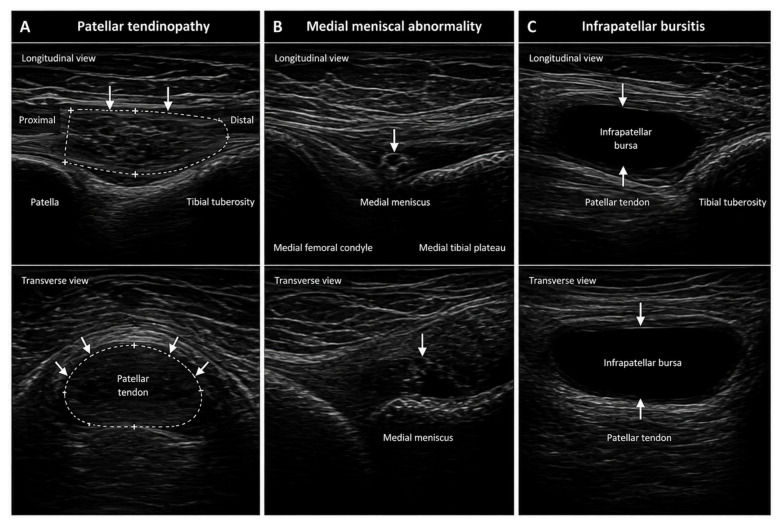
Representative ultrasonographic findings identified in the study population. (**A**) Patellar tendinopathy: longitudinal and transverse views demonstrating tendon thickening, focal hypoechogenicity, and loss of normal fibrillar architecture (arrows). (**B**) Medial meniscal abnormality: longitudinal and transverse views showing focal disruption and irregular morphology of the medial meniscus (arrows). (**C**) Infrapatellar bursitis: longitudinal and transverse views demonstrating abnormal anechoic fluid accumulation within the infrapatellar bursa (arrows).

**Figure 3:**
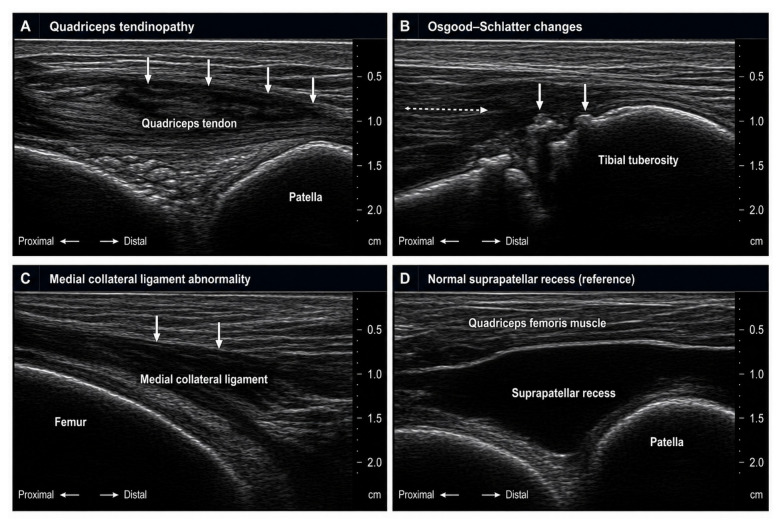
Additional representative ultrasonographic findings identified during the examination protocol. (**A**) Quadriceps tendinopathy demonstrating focal tendon thickening and hypoechogenicity (solid arrows). (**B**) Osgood–Schlatter changes involving the tibial tuberosity, with irregular ossification at the tibial tuberosity (solid arrows) and distal patellar tendon insertion (dashed arrow). (**C**) Medial collateral ligament abnormality demonstrating focal ligament thickening and altered echotexture (solid arrows). (**D**) Normal ultrasonographic appearance of the suprapatellar recess (reference image).

Representative ultrasonographic images illustrating the principal abnormalities identified in the study population are presented in Figure 2. Additional representative ultrasonographic images of less frequent abnormalities, together with a normal reference examination, are presented in Figure 3 to facilitate interpretation of the ultrasonographic findings described in the Results.

### 2.4. Pain Assessment

Knee pain intensity was assessed using the VAS, a widely used and validated instrument for the evaluation of subjective pain perception in clinical and sports medicine research [38,39,40,41,42].

Participants were asked to indicate their average knee pain intensity experienced during sports participation over the preceding week on a 10-cm horizontal line anchored by two extremes: 0 (“no pain”) and 10 (“worst imaginable pain”). The distance from the left endpoint to the participant’s mark was measured and recorded as the VAS score.

VAS scores were analyzed as continuous variables, with higher scores indicating greater pain intensity. The instrument was selected because of its simplicity, reliability, sensitivity to clinical changes, and widespread use in musculoskeletal research [38,39,40,43].

Pain assessments were performed immediately before the ultrasonographic examination to minimize potential bias arising from imaging findings. The obtained VAS scores were subsequently used for comparative analyses between ultrasonographic findings and as the dependent variable in the multivariable regression models.

### 2.5. Statistical Analysis

Statistical analyses were performed using IBM SPSS Statistics version 29.0 (IBM Corp., Armonk, NY, USA).

Continuous variables were assessed for normality using the Shapiro–Wilk test and are presented as mean ± standard deviation (SD). Categorical variables are presented as frequencies and percentages. For prevalence estimates, 95% confidence intervals (CIs) were calculated using the Wilson score method.

Prevalence estimates were calculated at the knee level (138 knees), whereas all inferential analyses, including comparisons of pain intensity, sport comparisons, odds ratio (OR) calculations, and multivariable regression analyses, were performed at the participant level (69 athletes). For all participant-level analyses, each athlete was classified as positive for a given ultrasonographic abnormality if the abnormality was identified in either knee. This approach avoided treating bilateral knee observations as statistically independent. Ultrasonographic abnormalities were not mutually exclusive, and individual athletes could present more than one abnormality. Therefore, each ultrasonographic finding was analyzed separately by comparing athletes with and without the corresponding abnormality. Therefore, clustered or mixed-effects statistical models were not required because all inferential analyses were performed using participant-level variables, with each athlete contributing a single observation. Although lower-limb dominance was recorded, it was not included in the primary statistical analyses because the study focused on participant-level associations between ultrasonographic abnormalities and pain intensity rather than side-specific differences between dominant and non-dominant limbs.

Comparisons between handball and basketball athletes were performed using independent-samples *t*-tests for normally distributed continuous variables and the χ^2^ test or Fisher’s exact test for categorical variables, as appropriate.

Differences in VAS pain scores were evaluated separately for each ultrasonographic abnormality by comparing athletes with and without the corresponding abnormality using the independent-samples *t*-test (or the Mann–Whitney U test when normality assumptions were not met). Effect sizes were calculated using Cohen’s d and interpreted according to conventional thresholds as small (0.2), moderate (0.5), and large (0.8).

ORs with corresponding 95% CIs were calculated to quantify the association between sport type and ultrasonographic abnormalities. Basketball athletes were used as the reference category.

To explore factors associated with pain intensity, an exploratory multivariable linear regression model was constructed using VAS pain score as the dependent variable. Independent variables entered into the model included age, BMI, sport type, previous knee injury, weekly training volume, and the most prevalent ultrasonographic abnormalities. Regression coefficients (β), standard errors (SEs), 95% CIs, and *p*-values were reported. Model performance was evaluated using the coefficient of determination (R^2^), adjusted R^2^, and the overall F-statistic.

Prior to regression analysis, multicollinearity was assessed using variance inflation factors (VIFs), while model assumptions were evaluated through inspection of residual plots and assessment of residual normality and homoscedasticity.

To assess the robustness of the primary findings, sensitivity analyses stratified by sport type were additionally performed using separate regression models for handball and basketball athletes.

A post hoc sample size analysis was performed using G*Power software (version 3.1.9.7, Heinrich Heine University Düsseldorf, Germany). Based on a medium effect size (f^2^ = 0.15), a significance level of 0.05, eight predictors in the multivariable regression model, and the available sample size of 69 athletes, the estimated statistical power was approximately 54%. Therefore, the multivariable regression model should be considered exploratory. Although multicollinearity and model assumptions were assessed and model diagnostics were satisfactory, the relatively small sample size may have reduced model stability and limited the ability to detect smaller associations. Consequently, the regression findings should be interpreted cautiously and require confirmation in larger prospective cohorts with adequate statistical power.

All statistical tests were two-tailed, and statistical significance was established at *p* < 0.05.

### 2.6. Ethical Considerations

The study was conducted in accordance with the ethical principles of the Declaration of Helsinki and was approved by the Ethics Committee of Pelican Clinical Hospital, Oradea, Romania (Approval No. 702/05 May 2025).

All participants received detailed information regarding the purpose and procedures of the study and provided written informed consent prior to enrollment. For participants younger than 18 years of age, written informed consent was additionally obtained from a parent or legal guardian.

Participation was voluntary, and participants were informed of their right to withdraw from the study at any stage without consequences. All collected data were anonymized before analysis to ensure participant confidentiality and compliance with applicable data protection regulations.

## 3. Results

### 3.1. Participant Characteristics

The study included 69 male athletes who underwent bilateral knee ultrasonographic assessment and completed the baseline questionnaire. The mean age of the participants was 20.8 ± 3.1 years, with the largest proportion of athletes aged 19–20 years. The cohort comprised 35 handball players (50.7%) and 34 basketball players (49.3%). Most participants resided in urban areas (78.3%), whereas 21.7% were from rural areas. Baseline demographic and training characteristics according to sport are presented in Table 1.

No statistically significant differences were observed between handball and basketball players regarding age, BMI, place of residence, training experience, weekly training volume, previous knee injury history, or limb dominance (all *p* > 0.05). Therefore, the two groups were considered comparable for subsequent analyses of ultrasonographic findings and clinical outcomes.

### 3.2. Ultrasonographic Findings

Bilateral knee ultrasonography revealed a range of structural abnormalities involving meniscal, ligamentous, tendinous, and periarticular structures. The prevalence of ultrasonographic findings for the right and left knees is presented in Table 2. Overall, tendon-related abnormalities and meniscal abnormality were among the most frequently observed findings, whereas collateral ligament abnormalities were less common.

Patellar tendinopathy was the most frequently detected ultrasonographic abnormality, affecting 21.0% of examined knees, followed by medial meniscal abnormality (15.9%) and infrapatellar bursitis (13.0%). In contrast, collateral ligament abnormalities were uncommon, with medial collateral ligament and lateral collateral ligament abnormalities accounting for 5.1% and 2.9% of cases, respectively.

A slightly higher prevalence of structural abnormalities was observed in the right knee compared with the left knee for most lesion categories. However, the differences between sides were generally small. Approximately 44.9% of athletes presented no detectable ultrasonographic abnormalities, whereas the remaining participants exhibited at least one structural alteration.

Overall, tendon-related abnormalities and meniscal abnormality constituted the predominant ultrasonographic findings within the study population.

### 3.3. Association Between Ultrasonographic Findings and Pain

#### 3.3.1. Prevalence of Asymptomatic Ultrasonographic Abnormalities

Among participants presenting ultrasonographic abnormalities, a proportion remained asymptomatic or reported only minimal pain (VAS ≤ 1). Patellar tendinopathy, medial meniscal abnormality, and infrapatellar bursitis were identified in several minimally symptomatic cases. These findings indicate that structural abnormalities may be detected in the absence of clinically significant participant-reported pain and suggest that ultrasonographic findings do not always correspond directly with symptom severity (Table 3).

Athletes with patellar tendinopathy, medial meniscal abnormality, and infrapatellar bursitis were significantly more likely to report symptomatic pain (VAS > 1) than participants without detectable ultrasonographic abnormalities. Nevertheless, a subset of athletes with these abnormalities remained asymptomatic or minimally symptomatic.

#### 3.3.2. Bilateral Versus Unilateral Ultrasonographic Abnormalities

Participants were classified as presenting unilateral abnormalities when structural abnormalities were identified in only one knee and bilateral abnormalities when similar lesions were identified in both knees.

To further evaluate the clinical relevance of structural knee abnormalities, participants were categorized according to lesion distribution as having no detectable lesion, unilateral lesions, or bilateral lesions. Mean VAS pain scores increased progressively across these categories, with the highest pain levels observed among athletes presenting bilateral abnormalities (Table 4).

Athletes with bilateral ultrasonographic abnormalities reported significantly greater pain intensity than those with unilateral lesions or no detectable abnormalities, suggesting that bilateral structural involvement may be associated with a greater symptom burden.

#### 3.3.3. Comparison of Pain Intensity According to Ultrasonographic Findings

Athletes with ultrasonographic abnormalities reported higher VAS pain scores than athletes without detectable ultrasonographic abnormalities. Among the evaluated ultrasonographic abnormalities, athletes with patellar tendinopathy exhibited the highest mean VAS pain scores, followed by those with medial meniscal abnormalities. In contrast, participants without detectable ultrasonographic findings generally reported minimal pain levels.

To further investigate these associations, pain intensity was compared between athletes with and without each ultrasonographic abnormality separately (Table 5).

Athletes with patellar tendinopathy reported significantly higher VAS pain scores than those without patellar tendinopathy (Cohen’s d = 1.24), indicating a large effect size. Similarly, athletes with medial meniscal abnormalities (Cohen’s d = 0.89) and infrapatellar bursitis (Cohen’s d = 0.73) also reported significantly higher pain scores than athletes without these ultrasonographic abnormalities. No statistically significant difference in pain intensity was observed for the remaining ultrasonographic abnormalities.

These findings indicate that the presence of patellar tendinopathy, medial meniscal abnormality, and infrapatellar bursitis is associated with higher pain intensity in young athletes, with patellar tendinopathy showing the strongest association.

### 3.4. Comparison of Ultrasonographic Findings Between Handball and Basketball Athletes

**Figure 4:**
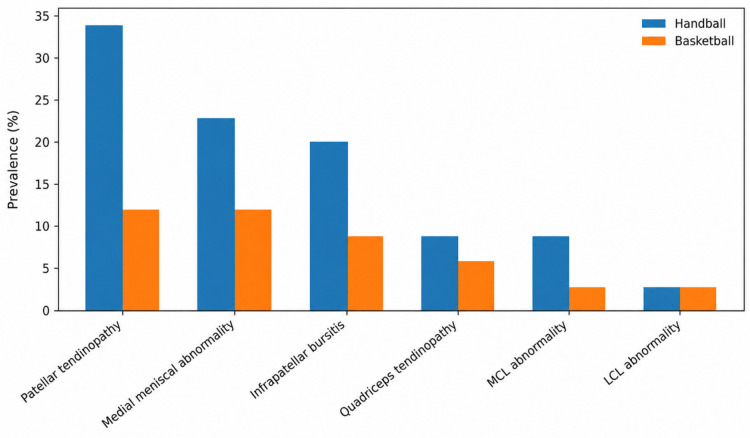
Comparison of the prevalence of ultrasonographic knee abnormalities between handball and basketball athletes.

The distribution of ultrasonographic findings according to sport is presented in Table 6 and Figure 4.

Handball athletes demonstrated a consistently higher prevalence of ultrasonographic abnormalities compared with basketball players. The most pronounced between-sport difference was observed for patellar tendinopathy, which occurred significantly more frequently among handball athletes than basketball players (34.3% vs. 11.8%; OR = 3.90, 95% CI: 1.09–13.95; *p* = 0.027). Although medial meniscal abnormality and infrapatellar bursitis were also more common among handball athletes, these differences did not reach statistical significance. No meaningful differences were observed for collateral ligament abnormalities.

Overall, these findings suggest that handball participation may be associated with a greater burden of tendon-related knee pathology, potentially due to the repetitive jumping, landing, and directional changes characteristic of the sport.

### 3.5. Multivariable Regression Analysis

To explore factors associated with knee pain, an exploratory multivariable linear regression model was constructed using VAS pain score as the dependent variable. Age, BMI, sport type, previous knee injury, weekly training volume, and the most prevalent ultrasonographic abnormalities were entered as independent variables (Table 7).

The multivariable regression model explained approximately 39% of the variance in VAS pain scores (R^2^ = 0.39; adjusted R^2^ = 0.34) and was statistically significant overall (F = 7.84, *p* < 0.001). After adjustment for age, BMI, sport type, previous knee injury, and weekly training volume, within this exploratory model, patellar tendinopathy demonstrated the strongest association with higher VAS pain scores (β = 1.34, *p* < 0.001). Within the exploratory multivariable model, medial meniscal abnormality (β = 0.70, *p* = 0.017) and infrapatellar bursitis (β = 0.54, *p* = 0.046) were also significantly associated with higher pain intensity.

Sport type, BMI, age, and weekly training volume were not independently associated with pain intensity after adjustment for the remaining variables. Previous knee injury demonstrated a borderline association with pain (β = 0.47, *p* = 0.051), although this did not reach statistical significance.

These findings suggest that ultrasonographic abnormalities, particularly patellar tendinopathy, were more strongly associated with pain intensity than demographic characteristics, previous knee injury, sport type, or weekly training volume.

**Figure 5:**
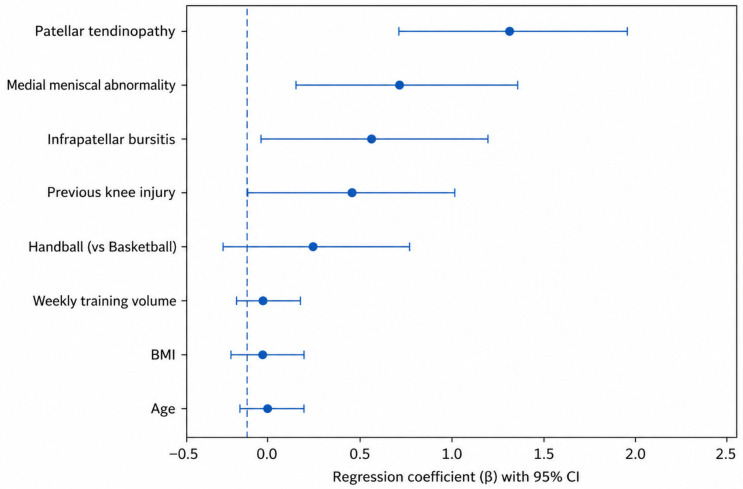
Forest plot of regression coefficients (β) and 95% CIs for independent correlates of VAS pain score. Positive coefficients indicate an association with increased pain intensity.

As illustrated in Figure 5, patellar tendinopathy demonstrated the strongest association with VAS pain score, followed by medial meniscal abnormality and infrapatellar bursitis. BMI, age, sport type, and weekly training volume showed weaker associations and were not statistically significant, while previous knee injury demonstrated a borderline association. The 95% CIs for patellar tendinopathy, medial meniscal abnormality, and infrapatellar bursitis did not cross the null value, supporting their independent associations with pain intensity.

### 3.6. Sensitivity Analysis Stratified by Sport

To assess the robustness of the observed associations, sensitivity analyses stratified by sport type were performed. Separate multivariable linear regression models were constructed for handball and basketball athletes, adjusting for age, BMI, previous knee injury, weekly training volume, and the most prevalent ultrasonographic abnormalities (Table 8).

Sensitivity analyses demonstrated generally similar patterns across both sports. After adjustment for age, BMI, previous knee injury, and weekly training volume, patellar tendinopathy remained the factor most strongly associated with VAS pain score in both handball and basketball athletes. Medial meniscal abnormalities were independently associated within the exploratory multivariable model, whereas infrapatellar bursitis remained significant only among handball athletes and showed a borderline association in basketball athletes. The magnitude and direction of the regression coefficients were broadly comparable between sports, indicating that the observed associations were not driven exclusively by one athletic discipline.

These findings support the robustness of the primary regression model and suggest that the observed associations between ultrasonographic abnormalities, particularly patellar tendinopathy, and pain intensity are generally consistent across both sports, although the subgroup analyses should be interpreted cautiously because of the limited sample size.

## 4. Discussion

The present study investigated the prevalence and clinical significance of ultrasonographic knee abnormalities in young male handball and basketball athletes. Several important findings emerged. First, patellar tendinopathy represented the most prevalent ultrasonographic abnormality, followed by medial meniscal abnormality and infrapatellar bursitis. Second, athletes presenting ultrasonographic abnormalities reported significantly higher pain scores than athletes without detectable lesions, with patellar tendinopathy demonstrating the largest clinical effect. Third, handball athletes exhibited a significantly greater prevalence of patellar tendinopathy compared with basketball athletes. Finally, multivariable regression analysis identified patellar tendinopathy as the strongest independent correlate of pain, followed by medial meniscal abnormality and infrapatellar bursitis. These findings largely support the study hypotheses and highlight the potential clinical relevance of ultrasonographic assessment.

### 4.1. Ultrasonographic Findings and Comparison with Previous Literature

Patellar tendinopathy was the most frequently identified abnormality in the present cohort. This finding is consistent with previous studies reporting a high prevalence of patellar tendon pathology among athletes participating in jumping sports, including handball, basketball, and volleyball [14,16,17]. The repetitive eccentric loading imposed on the extensor mechanism during jumping, landing, and rapid deceleration is believed to contribute to microstructural tendon degeneration and the subsequent development of tendinopathy [44,45,46].

The prevalence of medial meniscal abnormality observed in the present study was also consistent with previous reports describing meniscal pathology as a common finding among athletes exposed to repetitive rotational and pivoting movements [47,48,49]. Although meniscal abnormality is traditionally associated with acute trauma, repetitive mechanical loading may contribute to progressive degenerative changes even in relatively young athletic populations [8,48,50,51].

The interpretation of these findings, however, requires consideration of the inherent limitations of ultrasonographic assessment of intra-articular structures. Although musculoskeletal ultrasound may identify peripheral meniscal abnormalities, meniscal extrusion, contour irregularities, and focal hypoechoic defects, MRI remains the reference imaging modality for comprehensive evaluation of meniscal pathology. Consequently, the meniscal abnormalities reported in the present study should be interpreted as ultrasound abnormalities rather than definitive meniscal tears. Previous investigations have demonstrated variable diagnostic accuracy of ultrasonography for meniscal pathology, with greater accuracy reported for peripheral lesions and lower sensitivity for complex or deeply located tears [52,53,54,55,56]. Therefore, false-positive and false-negative findings cannot be excluded. Nevertheless, the observed association between ultrasound-detected meniscal abnormalities and pain intensity suggests that these findings may still represent clinically relevant structural alterations deserving further clinical evaluation. Future studies incorporating MRI confirmation would help clarify the diagnostic validity and clinical significance of these observations.

Interestingly, nearly half of the examined athletes presented no detectable ultrasonographic abnormalities. This observation highlights the heterogeneous nature of knee adaptations in sport and supports previous evidence suggesting that exposure to high training loads does not inevitably result in structural pathology [45,46,57,58]. It also reinforces the importance of considering both imaging findings and clinical presentation when evaluating athletes.

### 4.2. Relationship Between Ultrasonographic Findings and Pain

One of the most clinically relevant findings of the present study was the significant association between ultrasonographic abnormalities and pain intensity. Athletes presenting patellar tendinopathy, meniscal abnormality, or infrapatellar bursitis reported significantly higher VAS pain scores than athletes without detectable lesions.

Among all evaluated abnormalities, patellar tendinopathy demonstrated the strongest independent association with participant-reported pain after adjustment for age, BMI, sport type, previous knee injury, and weekly training volume. This finding is consistent with previous studies indicating that tendon pathology is frequently associated with activity-related pain, reduced functional capacity, and impaired sports performance [15,59,60]. The large effect size observed for patellar tendinopathy further suggests that the relationship is not only statistically significant but also clinically meaningful.

The findings also contribute to an ongoing debate within sports medicine regarding the relationship between imaging abnormalities and symptoms. In the present study, a subset of athletes demonstrated ultrasonographic abnormalities despite reporting minimal or no pain. This observation supports the concept that structural changes may exist in the absence of clinically relevant symptoms and highlights the importance of interpreting ultrasonographic findings within the broader clinical context. Several investigations have reported structural tendon changes in asymptomatic athletes, raising concerns regarding the clinical interpretation of imaging findings [6,21,61,62,63]. However, the present study demonstrated that specific abnormalities, particularly patellar tendinopathy, were strongly associated with pain intensity, supporting their clinical relevance in symptomatic athletes.

Furthermore, athletes presenting bilateral ultrasonographic abnormalities reported higher pain scores than those with unilateral lesions, suggesting that the overall structural burden may contribute to symptom severity. This finding supports the concept that the extent of structural involvement, rather than the mere presence of an abnormality, may influence the clinical manifestation of knee pain in young athletes.

### 4.3. Sport-Specific Differences

The comparison between sports revealed a significantly greater prevalence of patellar tendinopathy among handball athletes. Handball players were nearly four times more likely to present patellar tendinopathy than basketball players. This observation is biologically plausible and may be explained by differences in sport-specific movement patterns and mechanical loading characteristics.

Although both sports involve frequent jumping and landing, handball additionally requires repetitive high-intensity accelerations, abrupt directional changes, and physical contact, all of which may increase cumulative tendon loading [9,64,65,66]. Previous biomechanical studies have demonstrated substantial patellar tendon stress during handball-specific activities, supporting the hypothesis that handball athletes may be at greater risk of tendon-related pathology [15,67,68,69,70].

Interestingly, sport type itself did not remain an independent correlate of pain after adjustment for ultrasonographic abnormalities. This finding suggests that structural pathology may be more important than sport participation alone in explaining pain intensity. In other words, the higher pain observed among athletes was more closely associated with the presence of tendon or meniscal abnormalities than with sport participation alone.

### 4.4. Independent Correlates of Pain

The multivariable regression model demonstrated that patellar tendinopathy, medial meniscal abnormality, and infrapatellar bursitis were independently associated with higher pain scores.

These findings suggest that tendon pathology may be strongly associated with knee pain. Tendon degeneration is known to be associated with altered collagen organization, neovascularization, and nociceptive sensitization, which may contribute directly to symptom development [14,71,72,73,74]. Similarly, ultrasound-detected meniscal abnormalities and bursitis may increase local inflammatory activity and mechanical irritation, leading to pain during training and competition [75,76,77].

The sensitivity analyses stratified by sport produced comparable results, suggesting that the observed associations were robust and not driven exclusively by either handball or basketball athletes. Although these subgroup analyses should be interpreted cautiously because of the relatively small sample size, they strengthen confidence in the primary findings.

### 4.5. Clinical Implications

The findings of the present study have several practical implications for sports medicine clinicians, physiotherapists, athletic trainers, and coaches. First, musculoskeletal ultrasonography may be a useful tool for identifying tendon-related abnormalities in young athletes exposed to repetitive knee loading. Given its non-invasive nature, lack of ionizing radiation, relatively low cost, and ability to provide dynamic assessment, ultrasonography may have potential utility as an adjunct to clinical assessment of symptomatic athletes. However, the present cross-sectional findings should be considered hypothesis-generating and do not support the use of ultrasonography as a screening tool to identify athletes at increased risk of future symptoms or injury. Prospective longitudinal studies are required to establish its prognostic value.

The strong association observed between patellar tendinopathy and pain intensity suggests that tendon-related abnormalities should receive particular attention during clinical evaluation of athletes presenting with knee symptoms or persistent training-related pain. Detection of structural tendon changes associated with pain may assist clinicians in planning further clinical evaluation and rehabilitation. In this context, ultrasonography may assist clinicians in evaluating athletes presenting with knee pain and reduced sports participation.

From a practical perspective, athletes presenting with symptomatic patellar tendinopathy and corresponding ultrasonographic abnormalities may benefit from individualized load management strategies, progressive eccentric or heavy slow resistance training programs, neuromuscular control exercises, and regular clinical monitoring. Such interventions have demonstrated effectiveness in improving tendon function and reducing symptom severity in athletic populations. Emerging rehabilitation approaches, including blood flow restriction training in appropriately selected patients, may also represent useful adjuncts within individualized rehabilitation programs, although their role in the management of overuse tendon disorders requires further investigation [78,79]. Furthermore, the integration of ultrasonographic findings with clinical examination and functional assessment may support more personalized rehabilitation programs and return-to-sport decisions.

The significantly higher prevalence of patellar tendinopathy observed among handball athletes suggests that sport-specific injury prevention strategies remain important, although prospective longitudinal studies are needed to determine whether ultrasonographic findings predict future pain, functional limitation, or time-loss injury and whether they can be used to guide preventive interventions. Coaches and sports medicine professionals working with handball players should consider implementing structured injury prevention programs focusing on landing mechanics, lower-limb strength, movement quality, and training-load optimization. These interventions may help reduce cumulative tendon stress and potentially decrease the burden of overuse injuries throughout the competitive season.

Importantly, the present findings also emphasize that imaging findings should not be interpreted in isolation. Although ultrasonographic abnormalities were associated with higher pain scores, clinical decision-making should incorporate symptom severity, clinical examination, functional status, sport-specific workload, and individual athlete characteristics. A comprehensive approach combining imaging findings with clinical evaluation is likely to provide a more accurate assessment of an athlete’s current clinical status.

The present findings should be interpreted within the context of the study design. Although patellar tendinopathy, medial meniscal abnormality, and infrapatellar bursitis were significantly associated with higher pain intensity in the exploratory multivariable regression model, these associations should not be interpreted as evidence of causality or prognosis. The cross-sectional nature of the study does not allow determination of whether ultrasonographic abnormalities preceded the development of pain or whether they represent adaptive or incidental findings. Consequently, the present results should be considered hypothesis-generating rather than evidence supporting the use of ultrasonography as a screening tool to identify athletes at increased risk of future symptoms or injury. Prospective longitudinal studies are required to determine whether these ultrasonographic abnormalities predict future pain, functional limitation, or time-loss injury. Furthermore, because the regression model was developed using a relatively small sample with multiple explanatory variables, its findings should be considered exploratory and interpreted cautiously until confirmed in larger independent cohorts. Accordingly, imaging findings should not be interpreted as evidence of future injury risk on the basis of the present cross-sectional study. Furthermore, the presence of ultrasonographic abnormalities alone should not be considered an indication for treatment, as management decisions should be based on the overall clinical presentation, including symptoms, clinical examination, functional status, and sport-specific workload.

### 4.6. Strengths and Limitations

The present study possesses several methodological strengths. To our knowledge, relatively few investigations have simultaneously evaluated the prevalence of ultrasonographic knee abnormalities, their relationship with pain intensity, and potential sport-specific differences in young handball and basketball athletes. The inclusion of athletes from two sports characterized by high mechanical demands on the knee joint enabled the exploration of sport-related patterns of structural pathology.

Another important strength was the use of bilateral ultrasonographic examinations performed according to a standardized assessment protocol. This approach allowed comprehensive evaluation of multiple knee structures, including tendons, menisci, ligaments, and periarticular tissues. In addition, the integration of imaging findings with patient-reported pain outcomes enhanced the clinical relevance of the study and allowed assessment of whether structural abnormalities were associated with meaningful symptomatology rather than representing isolated imaging findings.

The statistical methodology also represents a strength of the present investigation. In addition to descriptive prevalence analyses, the study incorporated effect size estimation, multivariable regression modeling, OR calculations, and sensitivity analyses stratified by sport. These complementary analytical approaches provided a more comprehensive understanding of the observed relationships and strengthened the robustness of the findings.

Several limitations should nevertheless be acknowledged. First, the cross-sectional design precludes determination of temporal relationships and causal inferences between ultrasonographic abnormalities and pain. Consequently, the observed associations should not be interpreted as evidence that ultrasonographic abnormalities precede, predict, or increase the risk of future pain or injury. Likewise, the present findings do not support the use of musculoskeletal ultrasonography as a screening tool to identify athletes at increased risk of future symptoms. Prospective longitudinal studies are required to determine whether these ultrasonographic abnormalities predict future pain, functional limitation, or time-loss injury.

Second, the post hoc power analysis indicated that the multivariable regression model had limited statistical power, reflecting the relatively modest sample size. Consequently, the study may have been underpowered to detect smaller associations, particularly in the subgroup analyses. Larger cohorts would allow more precise estimation of prevalence rates, effect sizes, and factors independently associated with pain. Accordingly, the multivariable regression findings should be considered exploratory, as the relatively small sample size in relation to the number of predictors may have reduced model stability and increased the risk of overfitting. These findings therefore require confirmation in larger prospective cohorts.

Third, this was a single-center study involving athletes recruited from regional sports clubs. Consequently, the findings may not be fully generalizable to athletes from other geographic regions, competitive levels, or sporting environments. Multicenter studies including more diverse athletic populations are warranted to improve the external validity of the findings.

Fourth, only male athletes were included in the study. Consequently, the findings cannot be generalized to female athletes, who exhibit important differences in knee biomechanics, hormonal influences, injury mechanisms, and tendon adaptations.

Fifth, although ultrasonography offers numerous practical advantages, it possesses recognized limitations in the assessment of certain intra-articular structures. In particular, MRI remains the reference standard for comprehensive evaluation of meniscal pathology and other deep intra-articular abnormalities. Because MRI confirmation was not available for the meniscal abnormalities identified by ultrasonography, some ultrasound-detected meniscal abnormalities may have been misclassified. Although ultrasound can detect certain peripheral meniscal abnormalities, it is less accurate than MRI for comprehensive evaluation of intra-articular meniscal pathology. Consequently, the meniscal findings reported in the present study should be interpreted as ultrasound abnormalities rather than definitive meniscal tears.

Sixth, musculoskeletal ultrasonography is an operator-dependent imaging modality. Although all examinations were performed by a single experienced investigator using a standardized assessment protocol and excellent intraobserver agreement for the evaluated categorical ultrasonographic findings was demonstrated (κ = 0.84), interobserver reliability was not assessed. Consequently, the reproducibility of the ultrasonographic findings between different examiners cannot be determined and should be evaluated in future studies.

Seventh, pain intensity was assessed using the VAS; however, validated knee-specific functional outcome measures, such as the International Knee Documentation Committee (IKDC) score, Knee injury and Osteoarthritis Outcome Score (KOOS), Victorian Institute of Sport Assessment–Patella (VISA-P), or Kujala score, were not collected. In addition, objective functional assessments, including jump performance, return-to-sport status, activity limitation, or training modification, were not evaluated. Consequently, the relationship between ultrasonographic abnormalities, pain, functional impairment, and sports participation could not be comprehensively assessed.

Finally, several potentially relevant variables were not assessed, including objective training load, recent workload changes, competition exposure, playing position, playing level, training surface, footwear characteristics, lower-limb biomechanics, muscle strength, flexibility, and objective functional performance. These factors may influence both the development of ultrasonographic abnormalities and pain perception and therefore should be incorporated into future investigations to provide a more comprehensive understanding of factors associated with knee pathology in young athletes. Although lower-limb dominance was recorded, its relationship with the distribution of ultrasonographic abnormalities was not specifically evaluated because the primary analyses were conducted at the participant level rather than the limb level. In addition, the ultrasonographic protocol focused on the knee structures most commonly involved in sports-related overuse and traumatic injuries and did not include systematic assessment of all periarticular musculotendinous structures (e.g., the semimembranosus tendon). Consequently, additional musculotendinous abnormalities may have been present but were not evaluated.

### 4.7. Future Directions

The findings of the present study provide several avenues for future research. Most importantly, prospective longitudinal studies are needed to determine whether ultrasonographic abnormalities identified in asymptomatic or minimally symptomatic athletes are associated with subsequent pain development, functional impairment, reduced sports participation, or time-loss injuries, and whether they possess prognostic value.

Future studies should also incorporate knee-specific pain assessments to enable direct correlation between ultrasonographic findings and symptoms and to determine whether structural abnormalities identified on ultrasonography correspond to pain in the same knee. In addition, repeated ultrasonographic assessments throughout a competitive season would help distinguish transient physiological responses to training from progressive pathological changes. Longitudinal imaging could further clarify whether changes in ultrasonographic findings are accompanied by parallel changes in pain, function, or performance. Future studies should also include multiple independent sonographers and evaluate both intraobserver and interobserver reliability for categorical as well as quantitative ultrasonographic measurements, when applicable, to further strengthen the reproducibility and generalizability of ultrasonographic assessments. Furthermore, future investigations should combine imaging findings with validated knee-specific functional outcome measures (e.g., IKDC, KOOS, VISA-P, or Kujala score) together with objective functional assessments, including jump performance, return-to-sport status, and training modification, to provide a more comprehensive evaluation of the clinical relevance of ultrasonographic abnormalities.

Another important research direction involves the integration of imaging findings with objective biomechanical and workload metrics. Combining ultrasonography with measures such as jump performance, landing mechanics, strength testing, wearable sensor data, and external training loads may improve identification of athletes at increased risk of developing knee pathology. Such multidimensional approaches may ultimately support the development of individualized injury prevention strategies.

Future studies may also incorporate quantitative ultrasonographic techniques such as SWE to objectively assess biomechanical changes in periarticular knee structures. Previous studies have demonstrated the feasibility of SWE for measuring medial collateral ligament stiffness in healthy volunteers and for evaluating meniscal degeneration with MRI correlation. Combining conventional B-mode ultrasonography with SWE may therefore provide a more comprehensive assessment of both structural and mechanical tissue alterations in athletes and may improve understanding of early tissue adaptations associated with repetitive sports loading [80,81].

Future investigations should also evaluate female athletes, athletes from additional sports, larger multicenter cohorts, and more diverse competitive populations to improve the generalizability of the findings. Comparative studies involving different levels of competition may further clarify whether the prevalence and clinical significance of ultrasonographic abnormalities vary according to athletic exposure and performance demands and whether similar ultrasonographic abnormalities and their associations with pain are observed in female athletes.

Finally, studies comparing ultrasonography with MRI and comprehensive clinical assessment would help define the optimal role of ultrasound within sports medicine practice. Future studies incorporating MRI correlation are warranted to further validate the diagnostic accuracy and clinical significance of ultrasonographic findings, particularly for meniscal abnormalities. Determining the diagnostic accuracy, prognostic value, and cost-effectiveness of musculoskeletal ultrasonography may facilitate its evidence-based implementation in sports medicine practice.

## 5. Conclusions

The present study demonstrated that ultrasonographic knee abnormalities are common among young male handball and basketball athletes and are significantly associated with pain intensity. Patellar tendinopathy emerged as the most prevalent ultrasonographic finding and showed the strongest association with pain intensity. Ultrasonographic signs suggestive of medial meniscal abnormality and infrapatellar bursitis were also significantly associated with higher pain intensity within the exploratory multivariable model. Furthermore, handball athletes exhibited a higher prevalence of patellar tendinopathy than basketball athletes, suggesting potential sport-specific patterns of tendon loading.

These findings suggest that musculoskeletal ultrasonography may complement, but not replace, clinical assessment in the evaluation of symptomatic athletes. Because ultrasonography has recognized limitations in the assessment of intra-articular pathology, particularly the menisci, ultrasonographic meniscal findings should be interpreted as screening-level abnormalities rather than definitive meniscal tears, and MRI remains the reference imaging modality when comprehensive evaluation of intra-articular pathology is clinically indicated. The integration of ultrasonographic findings with clinical evaluation may facilitate the assessment of athletes presenting with knee pain and support targeted rehabilitation strategies. However, ultrasonographic findings should always be interpreted together with symptom severity, clinical examination, functional status, and sport-specific workload, and should not be used in isolation to guide clinical management or indicate treatment. Likewise, the presence of ultrasonographic abnormalities alone should not be considered an indication for intervention. Furthermore, because the present study was cross-sectional and the regression analysis was exploratory, prospective longitudinal studies are required to determine whether these ultrasonographic abnormalities have prognostic value for future pain, functional limitation, or time-loss injury.

## Figures and Tables

**Table 1:** Baseline characteristics of the study participants.

Variable	Handball (*n* = 35)	Basketball (*n* = 34)	*p*-Value
Age, years	21.1 ± 3.2	20.5 ± 3.0	0.48
BMI, kg/m^2^	23.6 ± 2.2	23.2 ± 2.0	0.44
Urban residence, *n* (%)	28 (80.0)	26 (76.5)	0.72
Rural residence, *n* (%)	7 (20.0)	8 (23.5)	0.72
Training experience, years	7.5 ± 3.5	6.9 ± 3.2	0.51
Weekly training volume, h/week	8.9 ± 2.9	8.3 ± 2.7	0.39
Previous knee injury, *n* (%)	10 (28.6)	8 (23.5)	0.63
Right dominant limb, *n* (%)	31 (88.6)	29 (85.3)	0.68

Note: Continuous variables are presented as mean ± SD. Categorical variables are presented as number (percentage). *p*-values were obtained using the independent samples *t*-test for continuous variables and the χ^2^ test for categorical variables.

**Table 2:** Prevalence of ultrasonographic knee abnormalities.

Ultrasonographic Finding	Right Knee *n* (%)	Left Knee *n* (%)	Total *n* (%)	95% CI (%)
Medial meniscal abnormality	12 (17.4)	10 (14.5)	22 (15.9)	10.8–23.7
Lateral meniscal abnormality	8 (11.6)	7 (10.1)	15 (10.9)	5.5–18.8
Patellar tendinopathy	16 (23.2)	13 (18.8)	29 (21.0)	14.2–30.6
Quadriceps tendinopathy	5 (7.2)	4 (5.8)	9 (6.5)	2.4–12.9
Infrapatellar bursitis	10 (14.5)	8 (11.6)	18 (13.0)	7.5–20.9
Medial collateral ligament abnormality	4 (5.8)	3 (4.3)	7 (5.1)	1.6–10.2
Lateral collateral ligament abnormality	2 (2.9)	2 (2.9)	4 (2.9)	0.4–7.2
Osgood–Schlatter changes	6 (8.7)	5 (7.2)	11 (8.0)	3.8–14.0
No abnormality detected	30 (43.5)	32 (46.4)	62 (44.9)	36.7–53.1

Note: Values are presented as *n* (%). The 95% CIs were calculated for the prevalence of each ultrasonographic finding using the Wilson score method.

**Table 3:** Distribution of asymptomatic and symptomatic cases according to ultrasonographic findings.

Ultrasonographic Finding	VAS ≤ 1 *n* (%)	VAS >1 *n* (%)	*p*-Value
No abnormality detected	25 (83.3)	5 (16.7)	Reference
Patellar tendinopathy	5 (17.2)	24 (82.8)	<0.001
Medial meniscal abnormality	4 (18.2)	18 (81.8)	0.002
Infrapatellar bursitis	3 (16.7)	15 (83.3)	0.005

Note: Asymptomatic/minimally symptomatic cases were defined as VAS ≤ 1, while symptomatic cases were defined as VAS > 1. Percentages were calculated within each ultrasonographic category. *p*-values were obtained using χ^2^ or Fisher’s exact tests, with the no-abnormality group used as the reference category.

**Table 4:** Pain intensity according to unilateral and bilateral ultrasonographic lesion patterns.

Lesion Pattern	*n* (%)	Mean VAS ± SD
No lesion	30 (43.5)	1.6 ± 0.8
Unilateral lesion	24 (34.8)	2.8 ± 1.0
Bilateral lesion	15 (21.7)	4.2 ± 1.3

Note: Values are presented as *n* (%) or mean ± SD. Participants were classified according to the presence of no lesion, unilateral lesions, or bilateral lesions. Comparisons of VAS pain scores were performed using one-way analysis of variance (ANOVA) with post hoc Bonferroni correction (or the Kruskal–Wallis test when assumptions were not met). Overall comparison (ANOVA): *p* < 0.001.

**Table 5:** Comparison of VAS pain scores according to the presence or absence of ultrasonographic abnormalities.

Ultrasonographic Finding	Present (Mean ± SD)	Absent (Mean ± SD)	*p*-Value	Effect Size (Cohen’s d)
Patellar tendinopathy	4.1 ± 1.3	2.0 ± 1.1	<0.001	1.24
Medial meniscal abnormality	3.2 ± 1.1	2.2 ± 1.2	0.014	0.89
Infrapatellar bursitis	2.8 ± 1.0	2.1 ± 1.2	0.038	0.73
Other abnormalities	2.5 ± 1.2	2.2 ± 1.1	0.071	0.42

Note: Pain scores were compared separately for each ultrasonographic abnormality between athletes with and without the corresponding finding using the independent-samples *t*-test (or the Mann–Whitney U test when normality assumptions were not met). Cohen’s d values of approximately 0.2, 0.5, and 0.8 represent small, moderate, and large effect sizes, respectively.

**Table 6:** Prevalence of ultrasonographic knee abnormalities in handball and basketball athletes.

Ultrasonographic Finding	Handball (*n* = 35)	Basketball (*n* = 34)	OR (95% CI)	*p*-Value
Medial meniscal abnormality	8 (22.9)	4 (11.8)	2.23 (0.58–8.54)	0.224
Lateral meniscal abnormality	5 (14.3)	3 (8.8)	1.72 (0.38–7.80)	0.481
Patellar tendinopathy	12 (34.3)	4 (11.8)	3.90 (1.09–13.95)	0.027
Quadriceps tendinopathy	3 (8.6)	2 (5.9)	1.50 (0.23–9.64)	0.669
Infrapatellar bursitis	7 (20.0)	3 (8.8)	2.58 (0.60–11.02)	0.186
Medial collateral ligament abnormality	3 (8.6)	1 (2.9)	3.10 (0.31–31.02)	0.308
Lateral collateral ligament abnormality	1 (2.9)	1 (2.9)	1.00 (0.06–16.64)	1.000

Note: ORs and 95% CIs were calculated using basketball athletes as the reference group. *p*-values were obtained using χ^2^ or Fisher’s exact tests, as appropriate. Percentages represent athletes with the ultrasonographic abnormality identified in at least one knee.

**Table 7:** Multivariable linear regression analysis examining factors independently associated with VAS pain score.

Predictor	β	SE	95% CI	*p*-Value
Age	0.06	0.04	−0.02 to 0.14	0.140
BMI	0.04	0.05	−0.06 to 0.14	0.430
Handball (vs Basketball)	0.31	0.22	−0.12 to 0.74	0.160
Previous knee injury	0.47	0.24	0.00 to 0.94	0.051
Weekly training volume	0.05	0.04	−0.03 to 0.13	0.220
Medial meniscal abnormality	0.70	0.29	0.13 to 1.27	0.017
Patellar tendinopathy	1.34	0.32	0.72 to 1.96	<0.001
Infrapatellar bursitis	0.54	0.27	0.01 to 1.07	0.046

Note: β = regression coefficient; CI = confidence interval; SE = standard error. Variables with *p* < 0.05 were considered statistically significant.

**Table 8:** Sensitivity analysis of the association between ultrasonographic findings and VAS pain score stratified by sport.

Predictor	Handball β (95% CI)	*p*	Basketball β (95% CI)	*p*
Age	0.05 (−0.05 to 0.15)	0.31	0.06 (−0.06 to 0.18)	0.28
BMI	0.03 (−0.08 to 0.14)	0.59	0.05 (−0.07 to 0.17)	0.41
Previous knee injury	0.44 (−0.10 to 0.98)	0.11	0.40 (−0.15 to 0.95)	0.15
Weekly training volume	0.04 (−0.06 to 0.14)	0.42	0.05 (−0.05 to 0.15)	0.33
Medial meniscal abnormality	0.74 (0.10 to 1.38)	0.025	0.62 (0.02 to 1.22)	0.043
Patellar tendinopathy	1.45 (0.67 to 2.23)	<0.001	1.21 (0.42 to 2.00)	0.004
Infrapatellar bursitis	0.57 (0.01 to 1.13)	0.047	0.47 (−0.03 to 0.97)	0.064

Note: Separate multivariable linear regression models were performed for handball and basketball athletes. Each model was adjusted for age, BMI, previous knee injury, weekly training volume, and the most prevalent ultrasonographic abnormalities. β = regression coefficient; CI = confidence interval. Due to the limited sample size within each sport subgroup, the sensitivity analyses should be interpreted as exploratory.

## Data Availability

The data supporting the findings of this study are not publicly available due to privacy and ethical restrictions involving human participants but are available from the corresponding author upon reasonable request.

## Abbreviations

The following abbreviations are used in this manuscript:

VAS	Visual Analogue Scale
BMI	Body Mass Index
ESSR	European Society of Musculoskeletal Radiology
MRI	Magnetic Resonance Imaging
ANOVA	Analysis of Variance
SD	Standard Deviation
CI	Confidence Interval
OR	Odds Ratio
SE	Standard Error
VIF	Variance Inflation Factor
SWE	Shear Wave Elastography
IKDC	International Knee Documentation Committee
KOOS	Knee injury and Osteoarthritis Outcome Score
VISA-P	Victorian Institute of Sport Assessment–Patella
